# Network Pharmacology-Based Investigation on Therapeutic Mechanisms of the *Angelica dahurica* Radix and *Ligusticum chuanxiong* Rhizoma Herb Pair for Anti-Migraine Effect

**DOI:** 10.3390/plants11172196

**Published:** 2022-08-24

**Authors:** Chu Duc Thanh, Chu Van Men, Hyung Min Kim, Jong Seong Kang

**Affiliations:** 1 College of Pharmacy, Chungnam National University, Daejeon 34114, Korea; 2 Institute of Biomedicine and Pharmacy, Vietnam Military Medical University, Hanoi 10000, Vietnam; 3 Clinical and Bioequivalence Testing Center, Vietnam Military Medical University, Hanoi 10000, Vietnam

**Keywords:** *Angelica dahurica*, *Ligusticum chuanxiong*, migraine, network pharmacology

## Abstract

Migraines are a common neurological disorder characterized by desperate throbbing unilateral headaches and are related to phonophobia, photophobia, nausea, and vomiting. The *Angelica dahurica* Radix and *Ligusticum chuanxiong* Rhizoma herb pair (ALHP) has been used to treat migraines for centuries in traditional Chinese medicine (TCM). However, the physiological mechanisms of migraine treatment have not yet been elucidated. In this study, a total of 50 hub targets related to the effect of 28 bioactive compounds in ALHP on anti-migraine were obtained through network pharmacology analysis. GO and KEGG analyses of the hub targets demonstrated that ALHP treatment of migraines significantly involved the G-protein-coupled receptor signaling pathway, chemical synaptic transmission, inflammatory response, and other biological processes. According to the degree of gene targets in the network, ACE, SLC3A6, NR3CI, MAPK1, PTGS2, PIK3CA, RELA, GRIN1, GRM5, IL1B, and DRD2 were found to be the core gene targets. The docking results showed a high affinity for docked conformations between compounds and predicted targets. The results of this study suggest that ALHP could treat migraines by regulating immunological functions, diminishing inflammation, and improving immunity through different physiological pathways, which contributes to the scientific base for more in-depth research as well as for a more widespread clinical application of ALHP.

## 1. Introduction

A migraine is a disabling primary headache that affects approximately 15% of the general population [1,2]. According to WHO reports [3], a migraine is ranked as the third most predominant medical disorder and the second neurological disease-induced disability worldwide [4,5]. The International Headache Society defines a migraine as an intensive throbbing headache that occurs with unilateral or bilateral localization [6]. Migraine Sphobia, nausea, and vomiting, as well as other neurological symptoms, such as tinnitus, dizziness, and cognitive impairment [7,8]. The principle treatment strategy for migraines aims to alleviate attack severity and duration, recover functioning ability, reduce the administration of medications, and expedite general management with minimal or no side effects [9,10]. In medication therapy for migraines, acute or abortive medications were usually prescribed for patients with infrequent migraine attacks, whereas the minority of preventive or prophylactic medications aimed to reduce the severity, duration or frequency of attacks in migraine patients [11]. Existing acute medications for migraines include non-steroidal anti-inflammatory drugs (NSAIDs), triptans (5-HT receptor agonists), calcitonin gene-related peptide (CGRP) receptor antagonists, and dopamine receptor antagonists [12]. In contrast, medications for migraine prophylaxis are categorized as beta-blockers, antidepressants, anticonvulsants, monoclonal antibodies against CGRP molecules, and receptors [12]. However, the unsatisfactory treatment efficacy of these medicines and their unpleasant adverse effects still requires prompt solutions [13]. Hence, there is an urgent demand for the discovery and development of novel and alternative migraine therapies to reduce the adverse effects of these medications. 

Based on the TCM theory, migraines belong to the category of disease resulting from “Head Wind”. In this regard, wind phlegm, deficiency syndrome, and blood stasis syndrome are also considered the primary pathophysiological mechanisms of migraines [14]. Therefore, the critical viewpoints of TCM practice for migraine treatment aims to extinguish wind, resolve phlegm, activate blood, and relieve stasis. ALHP was passed down as an ancient Chinese prescription called “Duliang”. In the folk Chinese medicine literature, Bai Yi Xuan Fang compiled by Wang Miu (1196 A.D), the Duliang prescription for headache treatment was fully recorded and described in terms of both formulation and usage [15]. The prescription consisted of *A. dahurica* radix and *L. chuanxiong* rhizoma with a weight proportion of 4:1. It has also been approved by the China State Food and Drug Administration (statement number Z20000011) to treat symptoms such as stuffy nose, runny nose, and headaches since 2000 [15]. The radix of *A. dahurica* Benth. et Hook. belongs to a perennial Apiaceae plant found abundantly in Taiwan, Korea, China, Japan, and Russia [16,17,18]. The antipyretic and analgesic properties of *A. dahurica* radix have been known for thousands of years [19]. This effect was proven to be based on the downregulation of the release of neurotransmitters and proinflammatory factors [20]. The rhizoma of *L. chuanxiong* Hort. is a commonly used traditional medicine for stimulating blood circulation and eliminating stasis in the clinical practice of TCM [21]. According to the approved Chinese Pharmacopoeia, *L. chuanxiong* rhizoma can be utilized to foster qi flow, blood circulation, wind expelling, and pain relief. It is often used to treat migraines, irregular menstruation, and rheumatism [22]. 

In TCM, the formula, which can be developed using one or several herbal components, contains many active ingredients. One ingredient might target one or multiple genes, proteins, and pathways in the pathogenic mechanism of a disease. Therefore, traditional herbal formulas can lead to an integrated or synergistic effect that is suitable for treating complex diseases. Network pharmacology is a recently developed method based mainly on the theory of systems biology and computer simulation technology [23]. The network pharmacology approach relies on the fundamental concept that multiple drugs in therapeutic fields act on multiple rather than single targets. By constructing the relationship between drugs, components, targets, and diseases, network pharmacology systematically investigates the multiple pharmacological effects of multiple components and multi-drug targets [24,25,26]. The investigation and analysis of the network of the interactions between multiple compounds, herbs, proteins, genes, and diseases, which is applied with network pharmacology approach, facilitates to elucidate the therapeutic efficacy of herbal formulas for disease [27,28].

In summary, this study aimed to exploit the network pharmacology approach to discover the potential bioactive compounds, core targets, and signaling pathways involved in the anti-migraine activity of ALHP. The results of this study may provide a theoretical basis for the molecular mechanism of ALHP in migraine treatment in future studies. 

## 2. Results

### 2.1. Identification of the Main Active Compounds and Corresponding Targets

In accordance with the cut-off criteria of OB ≥30% and DL ≥0.18, 24, the main compounds of ALHP were obtained from the TCMSP database, including 19 compounds of *A. dahurica* radix and 6 compounds of *L. chuanxiong* rhizoma (one overlapping compound, mandenol). Four other compounds, daucosterol [29], ferulic acid [30], ligustilide [31,32], and senkynolide A [33], which did not meet the filtering criteria but have potential bioactivities related to migraine, were selected as potential compounds for further experiments. A list of these compounds is provided in Table 1. Based on the SwissTargetPrediction database, after removing targets with a probability of less than 0.1 and duplicated targets between *A. dahurica* and *L. chuanxiong*, we obtained 655 targets corresponding to the compounds. The full names of the targets are displayed as gene symbols using the online UniProt database. The detailed data are shown in Table S1. 

### 2.2. Identification of Target Genes Related to Migraines and the Overlapping Targets

Removing the targets with a relevance score lower than twice the median in the GeneCards database and then the duplicated targets between the GeneCards, OMIM, and DisGeNet databases induced a total of 979 targets that were considered candidate therapeutic targets (Table S2). 

**Figure 1:**
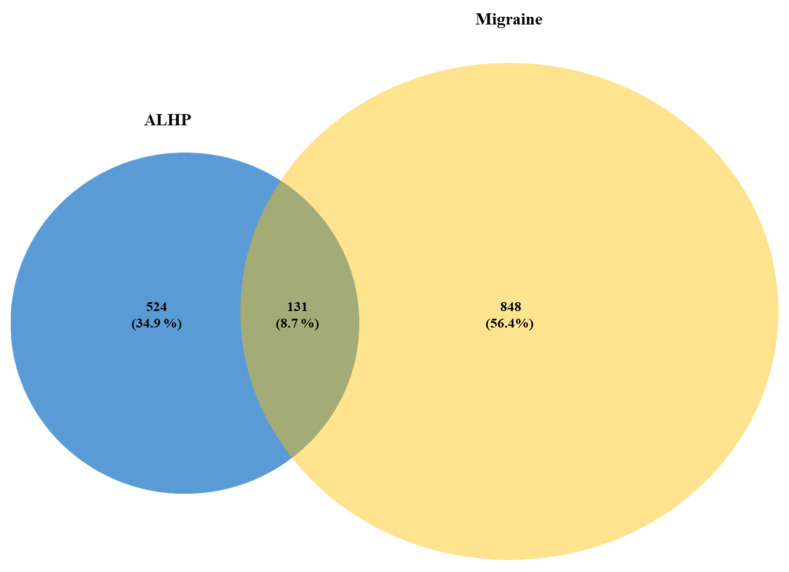
Venn diagram of the overlapping target of ALHP and migraines.

The targets of ALHP were intersected with those of migraines; 131 overlapping targets were determined (Table 2), and a Venn plot was drawn (Figure 1). These overlapping targets were considered potential targets in the therapeutic mechanism of ALHP against migraines.

### 2.3. Construction of a Herb–Compound–Target–Disease Network 

**Figure 2:**
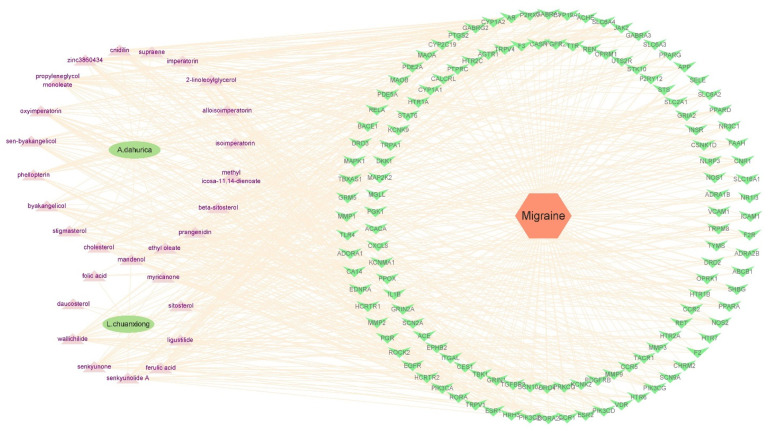
The herb–compound–target–disease network associated with ALHP and migraines.

The efficacy of TCM prescriptions underlies the synergistic effect of various compounds in different herbs on multiple targets involved in a disease. Insight into the effects of compounds in ALHP on the target proteins of migraines may help clarify the mechanism of the synergistic effect and the potential mechanism of ALHP for migraine treatment. Therefore, the herb–compound–target–disease network associated with ALHP and migraines was analyzed using the Cytoscape software (Figure 2). 

### 2.4. Establishing PPI Network of Overlapping Targets and Selection of Hub Targets 

**Figure 3:**
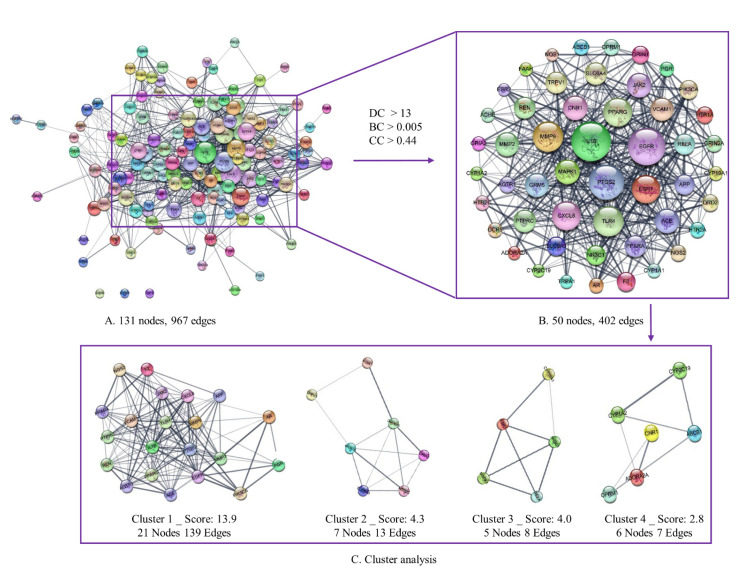
Identification of potential therapeutic targets for ALHP against migraines. (**A**) Construction of PPI networks of overlapping genes between ALHP and migraines via STRING. (**B**) Significant module determined via the function of topology analysis in Cytoscape. (**C**) Four clusters were predicted and visualized via the cluster analysis with MCODE algorithm (K-core threshold = 2). BC, betweenness centrality; CC, closeness centrality; DC, degree centrality.

The construction of the PPI network was implemented using the STRING web server. A total of 131 overlapping targets were entered into the STRING web server to yield a network with 131 nodes and 967 edges (Figure 3A). Thereafter, the PPI network was sent to Cytoscape and analyzed via topological analysis to further illustrate the hub targets of ALHP and migraines. The nodes with BC, DC, and CC values lower than the median value were removed. As a result, 50 nodes and 402 edges were identified in the PPI network (network parameters: degree > 13, betweenness centrality > 0.005, closeness centrality > 0.44), as shown in Figure 3B and Table S3. Finally, cluster analysis of these identified targets set up four clusters (Cluster 1: EGFR, PGR, JAK2, PIK3CA, AGTR1, IL1B, MMP9, PPARA, MMP2, NOS2, REN, APP, PTPRC, AR, ACE, VCAM1, PTGS2, TLR4, F2, PPARG, and CXCL8; Cluster 2: TRPV1, NOS1, GRIN2A, HTR2C, HTR2A, SLC6A3, GRIA2; Cluster 3: RELA, MAPK1, ESR1, NR3C1, CYP19A1; and Cluster 4: CYP2C19, CNR1, ABCB1, ADORA2A, CYP1A2, OPRM1), which may express the interconnectivity and function of clustered proteins (Figure 3C). Especially, the nodes of gene targets ACE, SLC6A3, NR3C1, and ABCB1 were determined as the seed nodes with the highest scoring node in clusters 1, 2, 3, and 4, respectively (Table S3). The seed node, calculated and predicted via MCODE algorithm, might become the key target with high-probability in the cluster [34]. This suggests that these genes may be crucial to the therapeutic treatment of migraines with ALHP.

### 2.5. GO Enrichment and KEGG Pathway Analysis

**Figure 4:**
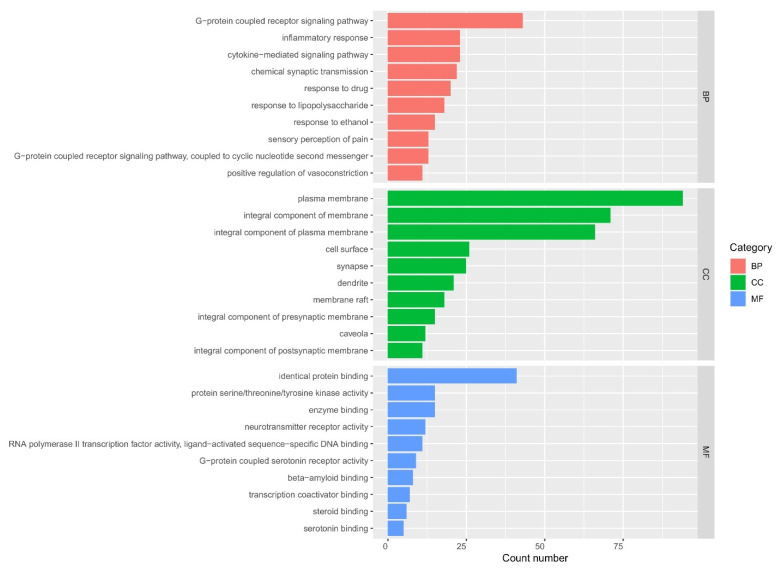
GO enrichment analysis for 50 key targets (BP: biological process; CC: cell component; MF: molecular function).

GO enrichment analysis was conducted using the DAVID web server to further elucidate the functions of the 50 hub genes. GO entries satisfying the criteria (*p* < 0.01, FDR < 0.05) included 218 biological processes, 46 cellular components, and 57 molecular functions (Table S4). The top 10 entries with the ordered -logP value in each category (BP, CC, and MF) were selected, which showed that the hub genes were substantially enriched in MF, such as identical protein binding, protein serine/threonine/tyrosine kinase activity, enzyme binding, and neurotransmitter receptor activity, and in CC, such as plasma membrane, integral component of membrane, integral component of plasma membrane, cell surface, and BP, such as G-protein-coupled receptor signaling pathway, inflammatory response, cytokine-mediated signaling pathway, and chemical synaptic transmission (Figure 4). 

**Figure 5:**
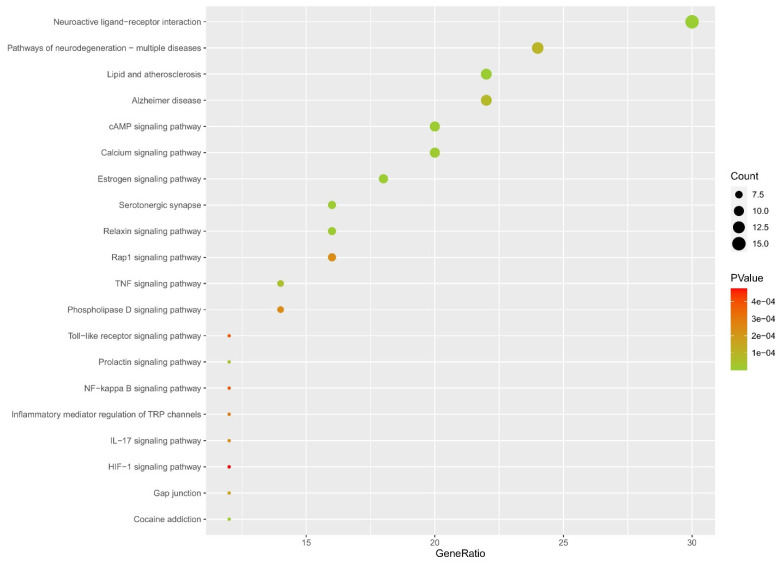
Top 20 KEGG pathways with ranked *p* value. The y-axis displays the KEGG pathway. The x-axis shows the amount of genes enriched in the pathway. The color represents the p-value, and the size of bubbles represents the amount of targets in the pathway. The color intensity from red to green shows the p value from high to low. The bigger the bubble size, the more targets in the pathway.

Sixty KEGG pathways (*p* < 0.01, FDR < 0.05) were obtained from the KEGG pathway analysis using DAVID. The elimination of obviously irrelevant KEGG pathways was performed, such as “pathways in cancer,” “Chagas disease,” and “hepatitis B”. The top 20 KEGG pathways with ranked *p* values were chosen and are shown in Figure 5 and Table S5. Accordingly, the potential KEGG pathways included neuroactive ligand–receptor interaction, pathways of neurodegeneration—multiple diseases, cAMP signaling pathway, calcium signaling pathway, estrogen signaling pathway, serotonergic synapse, Rap1 signaling pathway, TNF signaling pathway, inflammatory mediator regulation of TRP channels, NF-kappa B signaling pathway, Toll-like receptor signaling pathway, and HIF-1 signaling pathway. The top 20 KEGG pathways are listed in Table 3.

### 2.6. Construction of Gene Target—Pathway Network

**Figure 6:**
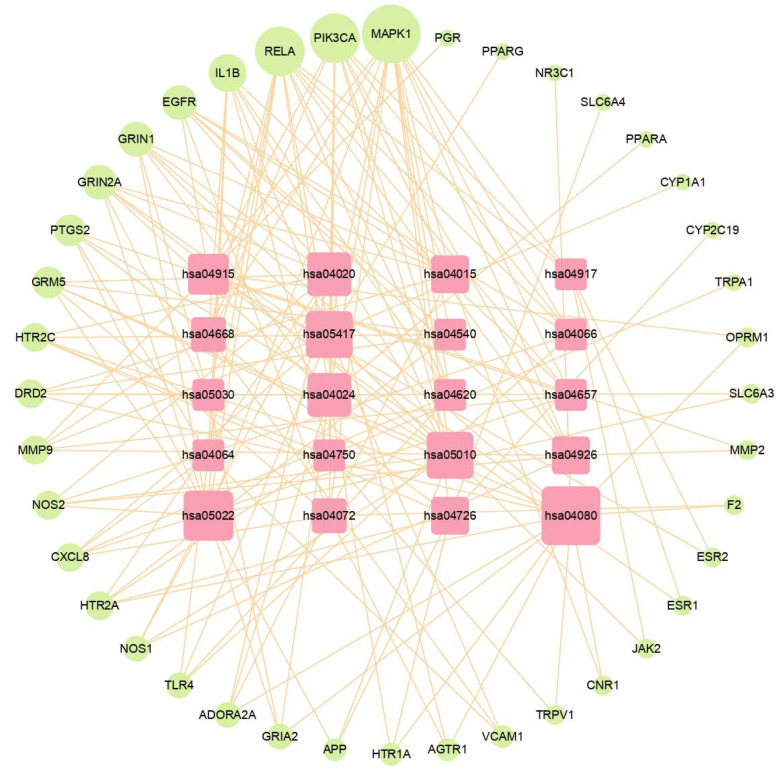
Gene target–pathway network of the ALHP against migraines. The light-yellow circles denote the target genes, and the pink squares denote pathways. Large size symbolizes larger degree.

Gene target–pathway network analysis was constructed based on the enriched pathways and corresponding gene targets that regulated these pathways, as shown in Figure 6. The relationships between the top 20 KEGG pathways and their regulated gene targets are presented in the diagram. According to the results of the network analysis, MAPK1 has the largest size; hence, it was considered the core gene target. In addition, other gene targets were relatively large, including RELA, PIK3CA, EGFR, NOS2, and DRD2. These gene targets were counted as potential key gene targets involved in the ALHP treatment of migraines.

### 2.7. Molecular Docking of the Bioactive Compounds of ALHP and Core Protein Targets 

**Figure 7:**
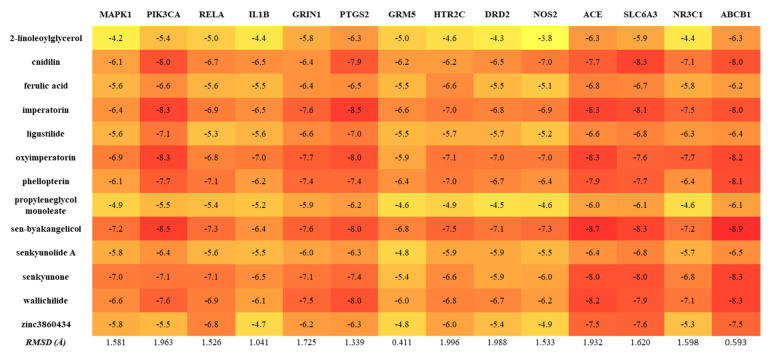
Heatmap of binding score (kcal.mol^−1^) between compound and protein target in the molecular docking study. The RMSD values below 2.0 Å indicate the docking results are acceptable.

**Figure 8:**
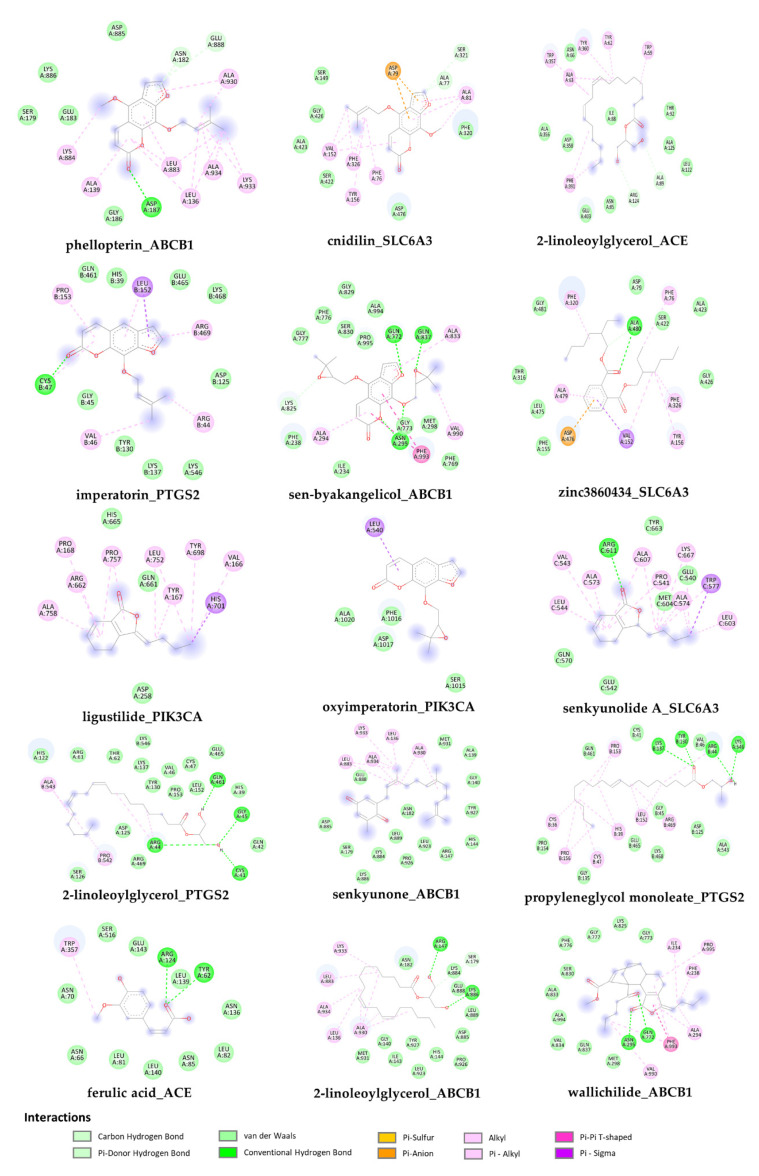
Two-dimensional visualization data of the compound–protein complex in the docking study.

Furthermore, compounds with high network connectivity are important in disease treatment. In accordance with the number of connected targets, there were 13 active compounds with high connectivity in the herb–compound–target–disease network used for the molecular docking assay (Figure 7). In addition, fourteen potential targets, which not only were considered as the seed node in PPI and cluster analysis but also have a high degree in the KEGG pathway–target network, were selected for docking study, including ACE (PDB ID: 1O86), SLC6A3 (UniProt ID: Q01959), NR3C1 (PDB ID: 6YMO), ABCB1 (PDB ID: 6FN1), MAPK1 (PDB ID: 3SA0), PIK3CA (PDB ID: 3ZIM), RELA (PDB ID: 4KV1), IL1B (PDB ID: 5R85), GRIN1 (PDB ID: 5KCJ), GRM5 (PDB ID: 6N4Y), PTGS2 (PDB ID: 5F19), DRD2 (PDB ID: 6CM4), HTR2C (PDB ID: 6BQG), and NOS2 (PDB ID: 3HR4). Among these target proteins, only the target SLC6A3 was modeled using the SWISS-MODEL and successfully validated via the Verify3D server (GMQE = 0.72; 87% of the residues with 3D-1D score ≥ 0.2). According to the binding energy in the docking assay, binding with a lower energy value is consistent with a stronger binding force to the protein. Generally, a binding capacity lower than −5.0 kcal/mol implies strong docking of conformation between ligand and protein, and lower values indicate stronger binding. The root mean square deviation (RMSD) values of the docking model for each compound were less than 2 Å, which confirmed that all the docking models were reliable [35]. Among the bioactive compounds, sen-byakangelicol, imperatorin, oxyimperatorin, cnidilin, ferulic acid, ligustilide, phellopterin, senkyunolide A, senkyunone, and wallichilide showed a high binding capacity for all 14 protein targets, whereas other compounds, such as zinc3860434, 2-linoleoylglycerol and propyleneglycerol monoleate, showed good interactions with only several targets (Figure 7). The detailed docking results of all 13 bioactive compounds with protein targets that showed the highest binding were visualized using PyMOL and Discovery Studio Visualizer software, as shown in Figure 8. 

## 3. Discussion

Migraines are one of the major causes of human disability worldwide [5]. To date, the clinical effectiveness of available remedies for migraine patients has been restricted due to poor efficacy, inescapable adverse effects, and medication abuse [13]. Thus, modern medicine still faces a huge challenge in the prevention and treatment of migraines. According to TCM principles, ALHP is effective in modulating qi flow, enhancing blood circulation, and soothing headaches. It has been commonly administered to alleviate different types of pain caused by qi and blood stasis conditions. Although ALHP manipulated in different dosage forms, such as pills, coated pills, and soft capsules, is broadly prescribed by TCM physicians, the pharmacodynamic material cause and mechanisms of action need more in-depth study.

Network pharmacology is a growing field that is widely applied in the field of drug discovery. This study employed integrated network pharmacology and molecular docking approaches to explore the molecular mechanisms of ALHP in migraine treatment. The findings showed that ALHP exerts a potential role in treating migraines by regulating multiple target genes, including ACE, SLC6A3, NR3C1, HTR2A, HTR2C, GRIN1, GRIN2A, DRD2, MAPK1, IL1B, RELA, NOS2, and PIK3CA.

The traditional use of *A. dahurica* radix as a remedy for headache and migraine has been documented in the folk literature and recent studies. The main chemical composition of *A. dahurica* includes coumarins with anti-oxidant and anti-inflammatory activities, namely imperatorin and oxyimperatorin, which are predicted to play key roles in migraine treatment [36,37]. In addition, phellopterin and cnidilin were detected as the major compounds in the TCM formula extract with anti-migraine activity [38]. The active ingredients in *L. chuanxiong* rhizoma, such as ferulic acid, senkyunone, ligustilide, and senkyunolide A, exhibited anti-inflammatory and anti-migraine activities and effectively prevented ischemic events [39,40]. 

Regarding the key targets, the angiotensin-converting enzyme (ACE) serves a primary role in stimulating inactive angiotensin I to active angiotensin II, a vasoconstrictor. Vasoconstrictor were early proved to cease migraine attacks [41,42]. The mitogen-activated protein kinase 1 (MAPK1), a member of the MAPK family, and MAP kinases are involved in many cellular signaling processes, such as proliferation and transcription regulation. Activated MAPK is proposed to modulate the synthesis and release of the neuropeptide calcitonin gene-related peptide (CGRP), which is associated with the pathogenesis of migraines [43,44]. In trigeminal ganglia neurons, MAPKs stimulate CGRP transcription via enhancer control [45]. Thus, the results of this study prove that MAPK1 targets mediating migraines via the MAPK signaling pathway, which is consistent with published studies. The increased level of peripheral proinflammatory cytokines involving IL1B enables an increase in the neuronal conduction of peripheral nociceptive neurons and, thus, a more significant peripheral nociceptive input, which may be attributed to central sensitization and improved hyperalgesia in the literature on chronic tension-type headaches [46]. In recently published studies, excessive serum levels of IL1B (proinflammatory cytokine) in patients suffering from migraines revealed that migraines had a tightened association with inflammation occurring within the peripheral endings of sensory neurons in the trigeminal ganglion system [47]. NR3C1 (glucocorticoid receptor) has effects on inflammatory responses, and especially has a wide distribution in neurons and neuroglia, which shows the active role of NR3C1 in migraines [48,49]. RELA was identified as a monomer in combination with other members of the Rel-like domain-containing proteins, such as RELB, NFKB1/p105, NFKB1/p50, REL, and NFKB2/p52, in order to form a homo- or heterodimeric complex of nuclear factor kappa B (NF-kappa-B). NF-kappa-B, a transcription factor involved in the inflammatory response, has been suggested as a mediator of the neurochemical cascade causing migraine attacks [50,51]. NOS enzymes, including NOS2, inhibit nitric oxide biosynthesis, thereby possibly functioning at peripheral locations to inhibit neurogenic dural vasodilation and at the endothelial level to hinder the dilation induced by CGRP [52]. PIK3CA functions as a catalytic subunit of phosphatidylinositol 3-kinase (PI3K), which phosphorylates signaling molecules through the PI3K pathway. In a rat model of migraines, activation of the PI3K/AKT signaling pathway may be triggered in the brain tissue [53]. Much preclinical and clinical evidence suggests that neurotransmitters and receptors, such as serotonin (5-HT), dopamine, and glutamate, are involved in migraine pathophysiology [54,55]. Consequently, the target SLC6A3 (dopamine transporter) fully participates in the pathogenesis of migraines. GRIN1, GRIN2A, and GRM5 are ionotropic and metatropic glutamate receptors, respectively. The development of glutamate receptor antagonists is one of the therapies for migraine treatment [56]. In addition, dopamine receptors play a significant role in migraine pathogenesis. A large number of studies have focused on the function of dopamine receptor D2 (DRD2) in central nervous system disorders, such as movement disorders, schizophrenia, migraine, and posttraumatic stress disorder [57,58]. Briefly, 5-HT and its receptors, such as HTR2A, HTR2C are implicated in migraines [54]. 

In terms of the pathway to further determine the therapeutic mechanisms of the ALHP formula, our study focused on the canonical KEGG pathways possibly linked to anti-migraine treatment and prophylaxis. In the serotonergic synapse pathway, the intracellular network cascade is triggered by serotonin, resulting in repressive or excitatory neurotransmission. The dispersion of serotonin receptors occurs in the brain, pain-signaling circuits, and cranial blood vessels. Anti-migraine therapies have been used to modulate serotonin receptors [55]. These pathways can be related to glial cell activation (neuroactive ligand-receptor interaction, cAMP signaling pathway, calcium signaling pathway, and gap junction), neuroinflammation (estrogen signaling pathway, NF-kappa B signaling pathway, TNF signaling pathway, Toll-like receptor signaling pathway, and Alzheimer’s disease pathway) [59], and neuro-immune responses (prolactin signaling pathway and cocaine addiction pathway) [60,61]. The Neuroactive ligand–receptor interaction signaling pathway is directly related to neurofunctions [62]. Neuroactive ligands binding to intracellular receptors affect neuronal function, which results from either binding transcription factors or regulating gene expression [63]. Neuroactive steroids act as hormones that regulate neurotransmitter receptors to either stimulate or inhibit neuronal activity [64]. It has been shown that the cAMP and possibly cGMP signaling pathway are associated with the activation of KATP channels. KATP channels are thought to be related to the pathophysiology of migraines through their function in the cerebral and meningeal arteries as well as the trigeminal system [65].

This study hypothesized that the anti-migraine effect of ALHP may be exerted mainly via the regulation of neuroactive ligand–receptor interaction, pathways of neurodegeneration—multiple diseases, serotonergic synapses, cAMP, and calcium signaling pathways. In addition, as holistic medicine, the anti-migraine mechanism of ALHP possibly acts through the NF-kappa B, TNF, cAMP, HIF-1, Toll-like receptor, and calcium signaling pathways to moderate the neurovascular systems and through neuro-inflammation and pain-related proteins, which produces a synergistic effect to relieve the burden of migraines.

## 4. Materials and Methods

**Figure 9:**
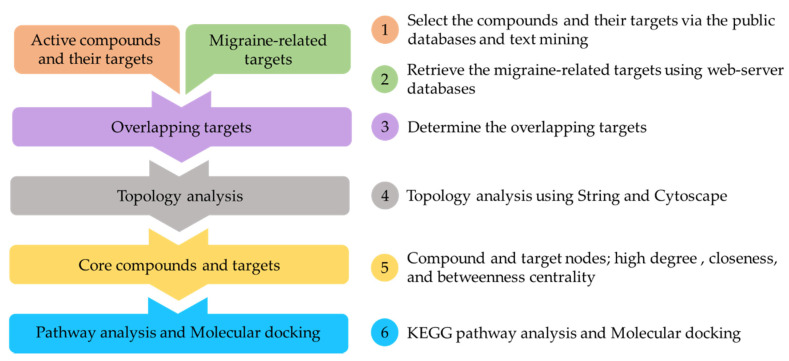
Workflow of network pharmacology analysis of ALHP.

The workflow of network pharmacology approach included these steps: (1) Compounds of ALHP were collected using the database of medicinal herbs and text mining. (2) Information about gene targets related to migraine disease was also retrieved in free and updated databases of human gene and diseases. (3) The overlapping targets were determined using a Venn diagram. (4) Topology analysis including the PPI analysis and network construction was carried out. (5) Core targets and compounds were screened and determined with threshold criteria of degree, closeness, and betweenness centrality. (6) GO and KEGG pathway analysis and molecular docking assay were performed on the potential compounds and core gene targets. The process of network pharmacology is described and summarized in Figure 9.

### 4.1. Collection of ALHP Active Compounds and Their Corresponding Targets 

First, the compounds of the two medicinal herbs in ALHP were collected from the TCM Systems Pharmacology Database (TCMSP, https://tcmspw.com/tcmsp.php (accessed on 02 November 2021)). Second, oral bioavailability (OB) and drug-likeness (DL) were utilized to select the potential active compounds, and their threshold values were set to OB ≥ 30% and DL ≥ 0.18, as previously described [66,67]. The OB of a drug is a major pharmacokinetic parameter that expresses the percentage of a drug dose in systemic circulation when administered orally [68]. DL properties are physicochemical properties that qualitatively assess the similarity between a compound and an existing or approved drug [69]. However, the published literature on the network pharmacology approach to the pharmacological mechanism of medicinal herbs has shown that herbal compounds had OB or DL values lower than threshold criteria but still participated in therapeutic mechanisms [70,71,72,73,74]. For that reason, the bioactive herbal compounds, reported in the title and abstract of papers in Pubmed and GoogleScholar with the searching query: “*Angelica dahurica*” or “*A. dahurica*” or “*Ligusticum chuanxiong*” or “*L. chuanxiong*” AND “migraine” or “headache”, were also collected. After combining and removing redundant compounds from two collection methods, the remaining compounds were selected for later steps. Finally, the most likely biological targets of the output compounds were acquired from the Swiss Target Prediction (http://swisstargetprediction.ch/ (accessed on 18 November 2021)) [75].

### 4.2. Collection of Migraine-Related Targets 

We collected targets related to migraines from three data sources: GeneCards (https://www.genecards.org (accessed on 20 November 2021)), DisGeNET (https://www.disgenet.org/home/ (accessed on 20 November 2021)), and OMIM (https://omim.org (accessed on 23 November 2021)). The keyword “migraine” was entered and searched for in each database. The GeneCards database, which was automatically mined and integrated from 150 web sources, provides user-friendly and comprehensive information regarding disease targets annotated and predicted in the human species. The wealth of GeneCards annotation was exploited with the GeneCards Inferred Functionality Score (GIFtS) algorithm to yield scores to predict the degree of functionality of the target. Based on the general criteria of GeneCards Inferred Functionality Score (GIFtS), the target with a score ≥30 was identified as the criteria target [76,77]. The Online Mendelian Inheritance in Man (OMIM) database, which is freely available and updated daily, contains information regarding known diseases and the corresponding genes in the genome of our species and the relationship between phenotype and genotype [78,79]. DisGeNET, a platform with comprehensive multifunctional data, integrates and processes information on human disorders and target genes to reveal the relationships between diseases and targets [80]. Combining targets obtained from the three databases and removing duplicates induced a set of potential targets associated with migraines.

### 4.3. Construction of Herb–Compound–Target–Disease Network and PPI Network

The overlapping targets from the two sets of targets of compounds and diseases were determined using the Venny tool (http://bioinfogp.cnb.csic.es/tools/venny/index.html (accessed on 25 November 2021)). The herb–compound–target–disease network was established and visually displayed using Cytoscape software (Cytoscape, Seattle, WA, USA, version 3.9.1, https://cytoscape.org/ (accessed on 26 August 2021)) with information input formats such as source node, target node, and source node attribute. 

The overlapping targets were imported into the STRING database (https://string-db.org/ (accessed on 14 December 2021)), and a protein–protein interaction (PPI) network was constructed with the following screening conditions: the species as “Homo sapiens”, the required interaction score at the level of medium confidence (0.400), and other parameters in default mode [81]. In the PPI plot, each node represents a gene, and the nodes are connected by edges. For further study of the PPI network, the PPI results in STRING were transferred to the Cytoscape software. The function “Analyze Network” in Cytoscape calculates the topological properties of a node in a network, namely degree centrality (DC), betweenness centrality (BC), and closeness centrality (CC). In addition, the app “ClusterViz” with the MCODE algorithm in Cytoscape was also used to clarify highly interconnected regions, or clusters, of the network, as well as to calculate and predict the seed node of cluster [34,82]. The default parameters optimized in the MCODE algorithm includes: Include Loop = false (off or unselected); Degree Threshold = 2; Haircut = true (on or selected); Fluff = false (off or unselected); NodeScore Threshold = 0.2; K-Core Threshold = 2; and MaxDepth = 100 [83,84].

### 4.4. Functional Enrichment Analysis of GO and KEGG Pathway

Gene Ontology (GO) functions and KEGG signaling pathways with potential targets were enriched using the Database for Annotation, Visualization, and Integrated Discovery (DAVID, https://david.ncifcrf.gov/ (accessed on 19 January 2022)) [85]. DAVID, an online bioinformatics resource, aims to interpret the functions of the submitted set of genes. In the DAVID analysis, the species as “Homo sapiens” was selected as the screening criterion. In addition, the dissimilarity in GO terms and KEGG signaling pathways with a false discovery rate (FDR) value of < 0.05 was considered significant. Finally, the bubble diagram of KEGG pathways was plotted using the ggplot2 package in the R language.

### 4.5. Molecular Docking of the Main Bioactive Compounds of ALHP and Core Target Proteins

A molecular docking study was performed to validate the association of compounds with key targets in the pathogenesis pathways in a network pharmacology study. The Avogadro program was utilized to form the 3D chemical structures of molecular ligands via the input of molecules in the SMILES format and auto-optimization function [86]. The three-dimensional (3D) structure of the protein receptor was obtained from the PDB online database (http://www.rcsb.org/ (accessed on 3 February 2022)). In another way, the 3D model of protein, based on the amino acid sequence from UniProt database [87] (https://www.uniprot.org/ (accessed on 2 December 2021)), was also built via the online server SWISS-MODEL (https://swissmodel.expasy.org/ (accessed on 3 February 2022)) and validated using the Verify3D Structure Evaluation Server (https://www.doe-mbi.ucla.edu/verify3d/ (accessed on 3 February 2022)) [87,88,89]. To remove molecular ligands and water from the protein receptor, the PyMol 2.4.0 program (https://pymol.org (accessed on 9 February 2022)) was utilized. The format of the receptor and ligand was transformed into pdbqt format via AutoDockTools 1.5.6 software. Active-binding pockets were identified. Subsequently, molecular docking was performed and calculated using Perl scripts in AutoDock Vina [90]. Finally, docking affinity was determined by selecting the affinity with the lowest binding energy, and the root mean square deviation (RMSD) values of all docked poses were measured by the RMSD/Superimpose function in AutoDock Tools. In data visualization, the 3D conformation structures of the ligands and receptors were displayed using PyMol software [91]. Discovery Studio Visualizer v21.1 software enabled the interaction between the protein and ligand to be visualized as a 2D image [92].

## 5. Conclusions

Using computational methods, including network pharmacology combined with molecular docking, this study revealed that the ALHP formula exerts an anti-migraine effect by regulating multiple targets and pathways in the pathogenesis of migraines. Among the components of the ALHP formula, imperatorin, ligustilide, oxyimperatorin, phellopterin, sen-byakangelicol, cnidilin, ferulic acid, senkyunolide A, senkyunone, and wallichilide were expressed in various associations in the pathophysiological pathways of migraines, which are considered as biomarkers of the formula. In addition, our study will provide a scientific basis for more comprehensive research and for a more widespread clinical application of ALHP in migraine treatment.

## Figures and Tables

**Table 1:** The selected active compounds of ALHP.

No.	Herb	MOL ID	Molecule Name	OB	DL	Smiles
1	*A. dahurica*	MOL003791	2-linoleoylglycerol	37.28	0.30	OCC(CO)OC(CCCCCCC/C=C\C/C=C\CCCCC)=O
2	*A. dahurica*	MOL001939	alloisoimperatorin	34.80	0.22	O=C1C=CC2=C(O)C3=C(OC=C3)C(C/C=C(C)\C)=C2O1
3	*A. dahurica*	MOL000358	beta-sitosterol	36.91	0.75	CCC(CCC(C)C1CCC2C1(CCC3C2CC=C4C3(CCC(C4)O)C)C)C(C)C
4	*A. dahurica*	MOL005800	byakangelicol	41.42	0.36	CC1(C(O1)COC2=C3C(=C(C4=C2OC(=O)C=C4)OC)C=CO3)C
5	*A. dahurica*	MOL000953	cholesterol	37.87	0.68	CC(C)CCCC(C)C1CCC2C1(CCC3C2CC=C4C3(CCC(C4)O)C)C
6	*A. dahurica*	MOL001956	cnidilin	32.69	0.28	O=C1C=CC2=C(OC/C=C(C)/C)C3=C(OC=C3)C(OC)=C2O1
7	*A. dahurica*	MOL002883	ethyl oleate	32.40	0.19	CCCCCCCC/C=C\CCCCCCCC(OCC)=O
8	*A. dahurica*	MOL001941	imperatorin	34.55	0.22	O=C1C=CC2=CC3=C(OC=C3)C(OC/C=C(C)/C)=C2O1
9	*A. dahurica*	MOL001942	isoimperatorin	45.46	0.23	O=C(C=C1)OC2=C1C(OC/C=C(C)/C)=C(C=CO3)C3=C2
10	*A. dahurica*	MOL007514	methyl icosa-11,14-dienoate	39.67	0.23	CCCCC/C=C/C/C=C/CCCCCCCCCC(OC)=O
11	*A. dahurica*	MOL013430	oxyimperatorin	43.60	0.29	O=C1C=CC2=CC3=C(OC=C3)C(OCC4C(C)(C)O4)=C2O1
12	*A. dahurica*	MOL002644	phellopterin	40.19	0.28	O=C1OC2=C(OC/C=C(C)/C)C3=C(C=CO3)C(OC)=C2C=C1
13	*A. dahurica*	MOL003588	prangenidin	36.31	0.22	O=C1C=CC2=C(C/C=C(C)\C)C3=C(OC=C3)C(O)=C2O1
14	*A. dahurica*	MOL005802	propyleneglycol monoleate	37.60	0.26	CCCCCCCCCC=CCCCCCCC(=O)OCCCO
15	*A. dahurica*	MOL005807	sen-byakangelicol	58.00	0.61	O=C1C=CC2=C(OCC3OC3(C)C)C4=C(OC=C4)C(OCC5OC5(C)C)=C2O1
16	*A. dahurica*	MOL000449	stigmasterol	43.83	0.76	CCC(C=CC(C)C1CCC2C1(CCC3C2CC=C4C3(CCC(C4)O)C)C)C(C)C
17	*A. dahurica*	MOL001506	supraene	33.55	0.42	C/C(CC/C=C(C)/CC/C=C(C)/C)=C\CC/C=C(C)\CC/C=C(C)/CC/C=C(C)/C
18	*A. dahurica*	MOL001749	zinc3860434	43.59	0.35	CCCCC(CC)COC(=O)C1=CC=CC=C1C(=O)OCC(CC)CCCC O=C(C1=CC=CC=C1C(OC[C@H](CC)CCCC)=O)OC[C@H](CC)CCCC
19	*A. dahurica/* *L. chuanxiong*	MOL001494	mandenol	42.0	0.19	CCCCC/C=C\C/C=C\CCCCCCCC(OCC)=O
20	*L. chuanxiong*	MOL000433	folic acid	69.0	0.71	C1=CC(=CC=C1C(=O)NC(CCC(=O)O)C(=O)O)NCC2=CN=C3C(=N2)C(=O)N=C(N3)N
21	*L. chuanxiong*	MOL002135	myricanone	40.6	0.51	COC1=C(OC)C(O)=C2CCCCC(CCC3=CC(C1=C2)=C(O)C=C3)=O
22	*L. chuanxiong*	MOL002151	senkyunone	47.7	0.24	O=C1C(C)=CC(C=C1C/C=C(C)/CC/C=C(C)/CC/C=C(C)\C)=O
23	*L. chuanxiong*	MOL000359	sitosterol	36.9	0.75	CCC(CCC(C)C1CCC2C1(CCC3C2CC=C4C3(CCC(C4)O)C)C)C(C)C
24	*L. chuanxiong*	MOL002157	wallichilide	42.3	0.71	CCCCC(=O)C12CCC(C=C1C(=O)OC)C3C2C4=C(CC3)C(=CCCC)OC4=O
25	*L. chuanxiong*	MOL000085	daucosterol	20.63	0.63	CCC(CCC(C)C1CCC2C1(CCC3C2CC=C4C3(CCC(C4)OC5C(C(C(C(O5)CO)O)O)O)C)C)C(C)C
26	*L. chuanxiong*	MOL000360	ferulic acid	55.14	0.06	COC1=CC(/C=C/C(O)=O)=CC=C1O
27	*L. chuanxiong*	MOL002201	ligustilide	51.3	0.07	CCCC=C1C2=C(C=CCC2)C(=O)O1
28	*L. chuanxiong*	MOL002208	senkynolide A	26.6	0.07	CCCCC1C2=C(C=CCC2)C(=O)O1

**Table 2:** The overlapping targets between ALHP and migraines.

CA14	PGR	STAT6	HTR2A	ICAM1	PDE2A	SLC6A3	HTR1B
AR	PPARA	TRPM8	HTR6	MMP9	PDE5A	CSNK1D	NOS1
SHBG	PPARD	TYMS	HTR7	SELE	PIK3CB	MAPK1	CALCRL
RORA	PPARG	ACHE	MAOB	TRPV1	TBXAS1	P2RY12	CCR2
ESR1	PTGS2	CHRM2	MMP1	UTS2R	VCAM1	PIK3CA	PPOX
ESR2	ROCK2	NOS2	MMP3	MMP2	BACE1	SCN10A	OPRK1
FAAH	ADORA1	NR1I3	SCN9A	APP	CYP1A1	SCN2A	OPRM1
CYP19A1	DRD3	SLC6A4	SLC2A1	KCNK2	CYP1A2	STK10	CXCL8
CYP2C19	EGFR	VDR	TACR1	TLR4	F3	TRPA1	FGFR2
SLC6A2	EPHB2	ADRA1B	TGFBR2	ADRA2B	RELA	ACE	GRIA2
ADORA2A	JAK2	DRD4	ABCB1	CCR5	SLC16A1	AGTR1	GRIN1
CASR	KCNMA1	GABRA3	ACACA	CES1	TTR	KCNK9	GRIN2A
CNR1	MAP2K2	GABRA5	DKK1	EDNRA	CCR1	TRPV4	ITGAL
F2R	PIK3CD	GABRG2	DRD2	IL1B	F2	STS	PGK1
HRH3	PIK3CG	GRM5	HTR1A	MGLL	MAOA	PTPRC	REN
NLRP3	PRKCG	HCRTR1	HTR2C	P2RX7	PDGFRB	INSR	TBK1
NR3C1	RET	HCRTR2					

**Table 3:** Target genes in the top 20 of KEGG pathways.

ID	Term	*p*-Value	Genes
hsa04080	Neuroactive ligand–receptor interaction	1.14 × 10^−8^	GRIA2, HTR1A, HTR2C, TRPV1, HTR2A, OPRM1, F2, NR3C1, GRIN1, GRM5, GRIN2A, ADORA2A, CNR1, AGTR1, DRD2
hsa05022	Pathways of neurodegeneration—multiple diseases	9.62 × 10^−5^	GRIA2, APP, GRM5, GRIN2A, NOS2, IL1B, MAPK1, NOS1, PTGS2, RELA, SLC6A3, GRIN1
hsa05417	Lipid and atherosclerosis	4.62 × 10^−7^	VCAM1, CXCL8, PIK3CA, IL1B, CYP1A1, MAPK1, PPARG, JAK2, TLR4, MMP9, RELA
hsa05010	Alzheimer disease	7.87 × 10^−5^	APP, GRM5, GRIN2A, PIK3CA, NOS2, IL1B, MAPK1, NOS1, PTGS2, RELA, GRIN1
hsa04024	cAMP signaling pathway	5.59 × 10^−6^	GRIA2, GRIN2A, ADORA2A, PIK3CA, HTR1A, MAPK1, DRD2, PPARA, RELA, GRIN1
hsa04020	Calcium signaling pathway	1.09 × 10^−5^	GRM5, GRIN2A, ADORA2A, NOS2, AGTR1, HTR2C, NOS1, HTR2A, EGFR, GRIN1
hsa04915	Estrogen signaling pathway	1.43 × 10^−6^	PIK3CA, MMP2, MAPK1, PGR, OPRM1, ESR1, MMP9, EGFR, ESR2
hsa04726	Serotonergic synapse	5.03 × 10^−6^	APP, HTR1A, MAPK1, HTR2C, HTR2A, CYP2C19, PTGS2, SLC6A4
hsa04926	Relaxin signaling pathway	1.08 × 10^−5^	PIK3CA, NOS2, MMP2, MAPK1, NOS1, MMP9, RELA, EGFR
hsa04015	Rap1 signaling pathway	2.40 × 10^−4^	GRIN2A, ADORA2A, PIK3CA, CNR1, MAPK1, DRD2, EGFR, GRIN1
hsa04668	TNF signaling pathway	5.22 × 10^−5^	VCAM1, PIK3CA, IL1B, MAPK1, PTGS2, MMP9, RELA
hsa04072	Phospholipase D signaling pathway	2.44 × 10^−4^	GRM5, CXCL8, PIK3CA, AGTR1, MAPK1, F2, EGFR
hsa05030	Cocaine addiction	1.02 × 10^−5^	GRIA2, GRIN2A, DRD2, RELA, SLC6A3, GRIN1
hsa04917	Prolactin signaling pathway	5.88 × 10^−5^	PIK3CA, MAPK1, JAK2, ESR1, RELA, ESR2
hsa04540	Gap junction	1.75 × 10^−4^	GRM5, MAPK1, HTR2C, HTR2A, DRD2, EGFR
hsa04657	IL-17 signaling pathway	2.39 × 10^−4^	CXCL8, IL1B, MAPK1, PTGS2, MMP9, RELA
hsa04750	Inflammatory mediator regulation of TRP channels	2.91 × 10^−4^	PIK3CA, TRPA1, IL1B, HTR2C, TRPV1, HTR2A
hsa04064	NF-kappa B signaling pathway	3.83 × 10^−4^	VCAM1, CXCL8, IL1B, PTGS2, TLR4, RELA
hsa04620	Toll-like receptor signaling pathway	3.83 × 10^−4^	CXCL8, PIK3CA, IL1B, MAPK1, TLR4, RELA
hsa04066	HIF-1 signaling pathway	4.76 × 10^−4^	PIK3CA, NOS2, MAPK1, TLR4, RELA, EGFR

## Data Availability

Data are contained within the article and Supplementary Material.

## Supplementary Materials

The following supporting information can be downloaded at: https://www.mdpi.com/article/10.3390/plants11172196/s1; Table S1. Compound Targets of *A. dahurica*, *L. chuanxiong* and ALHP; Table S2. Targets of Migraine; Table S3. Hub targets and Clusters in PPI analysis; Table S4. GO analysis; Table S5. KEGG analysis.
